# The Winthir Collection: An Identified Historical Skeletal Series From Munich, Germany

**DOI:** 10.1002/ajpa.70302

**Published:** 2026-06-25

**Authors:** Ja Young Lee, Barbara Schratzenstaller, Elsa Seyr, Maren Velte, Kristin von Heyking, Bernd Trautmann, Eva Kropf, Jochen Haberstroh, Albert Zink, Christof Paulus, Michaela Harbeck

**Affiliations:** ^1^ Bavarian State Collections of Natural History (SNSB), state Collection for Anthropology Munich (SAM) Munich Germany; ^2^ Faculty of History and Art History, Institute of Bavarian History Ludwig‐Maximilians‐University of Munich Munich Germany; ^3^ AnthroArch GbR Grafrath Germany; ^4^ Bavarian Office for the Conservation of Historical Monuments Munich Germany

**Keywords:** documented skeletal collection, nineteenth‐century Europe, non‐adult growth and development, rural‐to‐urban migration

## Abstract

Documented skeletal collections that can be linked to individual life‐history information remain critical resources for bioarchaeological research, yet nonmodern identified series are still rare in Central Europe. We present the Winthir Collection, a late‐19th‐century skeletal assemblage from Munich, Germany, comprising 245 identified individuals recovered from an abandoned cemetery extension.

Individual attribution was achieved through the integration of osteological analysis with grave registry data and archival sources. For most individuals, reconstructed profiles include age at death, place of birth, family relations, marital status, occupation, and recorded cause of death, allowing skeletal observations to be interpreted within a documented biographical framework.

The assemblage contains a substantial number of non‐adults (*n* = 90), markedly expanding the limited pool of identified non‐adult remains available for the region. Preservation is generally good, and skeletal evidence of medical intervention—including cranial procedures consistent with autopsy—can be evaluated alongside documentary information. Curated at the State Collection for Anthropology Munich, the Winthir Collection offers a rare opportunity to examine osteological variation within a historically documented population and provides an important reference dataset for future methodological work, particularly in the study of non‐adult remains.

## 1 Introduction

Documented human osteological collections are fundamental resources for archeology, biological anthropology, and forensic science (e.g., Henderson and Alves‐Cardoso 2018; Mann et al. 2021). Such identified reference collections contain skeletal remains of individuals with known biographical details and provide essential datasets for validating and developing methods used to reconstruct biological profiles (Hens et al. 2008; Lesciotto and Klales 2024; Marino et al. 2021; Rissech et al. 2012). They allow population‐specific calibration of osteological techniques, support research on growth and development when non‐adult skeletons are available, and enable more precise interpretation of pathological and occupational markers in individuals with documented medical or occupational histories (e.g., Karakostis and Hotz 2024; Pietrobelli et al. 2022; Simon et al. 2024; Villotte and Santos 2023).

Documented osteological collections are commonly categorized as either historical or nonmodern (generally pre–World War II) and modern or contemporary (post–World War II). This distinction reflects substantial changes in nutrition, health care, living conditions, and population structure during the 20th century, which led to pronounced secular and epidemiological shifts with measurable biological consequences (Dirkmaat et al. 2008; Langley and Jantz 2020; Marinho et al. 2018; Henderson and Alves‐Cardoso 2018; Santos 2020). As a result, pre‐WWII collections are generally unsuitable as direct forensic reference samples for contemporary populations. At the same time, they remain essential comparative datasets for the study of nonmodern populations and for reconstructing life conditions prior to the major demographic and epidemiological transitions of the 20th century.

Despite their significance, identified skeletal series remain unevenly distributed geographically. Numerous well‐known collections exist, for example, in Portugal and Italy, and several internationally prominent reference collections—such as the Terry and Hamann–Todd Collections in the United States and the Spitalfields Collection in the United Kingdom—are widely cited in methodological research (Alves‐Cardoso and Campanacho 2022; Hunt and Albanese 2005; Molleson and Cox 2002). In contrast, nonmodern identified skeletal collections from Central Europe are comparatively scarce (Petaros et al. 2021; see Supporting Information I).

Within this context, we introduce the Winthir Collection, a documented late nineteenth‐ and early twentieth‐century skeletal assemblage from Munich, southern Germany. The collection was formed through the retrospective identification of individuals buried in the abandoned eastern extension of the Winthir Cemetery in Munich, Bavaria, Germany. Following archaeological recovery, individual attribution is achieved by systematically integrating osteological assessment with grave registry data and extensive archival research.

The Winthir Collection has recently been documented in an interdisciplinary monograph that provides detailed archaeological, historical, ethical, and catalog‐based information on the collection and its identified individuals (Harbeck and Paulus in press). The aim of the present contribution is different. Rather than providing comprehensive documentation of the assemblage, it introduces the collection as a bioanthropological research resource and presents a series of population‐level syntheses that demonstrate its scientific potential. These include analyses of demographic structure, migration histories, occupational composition, mortality patterns, preservation characteristics, trauma prevalence, evidence of medical intervention, and the occurrence of ossified tissues. Particular attention is given to the collection's significance as a documented non‐adult skeletal series and its value for future methodological and comparative research.

## 2 Historical and Archaeological Context of the Winthir Cemetery

The Winthir Cemetery is located in Munich's Neuhausen district and surrounds the Catholic filial church “Mariä Himmelfahrt.”

In 2014, construction work adjacent to the current cemetery uncovered human remains belonging to an eastern extension abandoned in the early 20th century. Archaeological excavations in 2014 and 2018 documented approximately 230 grave features containing more than 245 individuals. Most grave features contained a single articulated burial, whereas approximately 20 contained multiple superimposed burials. In addition, numerous disarticulated skeletal remains recovered from grave backfill deposits could be reconstructed into further individuals.

The excavated section also included a designated non‐adult burial area, which is particularly relevant for the composition of the assemblage (Figure S2). Individuals were generally interred in an extended supine position, oriented east–west and placed in nailed wooden coffins. Grave depths averaged 1.8 for adults and 1.3 m for non‐adults under 12 (Harbeck and Paulus in press).

As expected in cemeteries with reusable grave plots, many pits also contained redeposited skeletal remains within grave backfill deposits. These remains originated from earlier burials disturbed during the reopening and reuse of grave plots and subsequently redeposited within the soil used to refill the grave.

Grave goods such as buttons, bodice hooks, shoes, and hairpins indicate burial in ceremonial or everyday clothing.

Historical documents from the city administration indicate that the excavated section of the cemetery was mainly used at the end of the 19th century. This period falls within the Prince Regent era (1886–1912), during which the Kingdom of Bavaria underwent accelerated socioeconomic change, transitioning from a predominantly agrarian to a partially industrialized society. Munich expanded rapidly during this time, incorporating formerly independent rural suburbs such as Neuhausen. Around 1900, Neuhausen was characterized as a predominantly lower‐class borough shaped by industrial and military functions, with over half of the population employed as trade assistants or day laborers. Migration drove rapid population growth, increasing from 784 inhabitants in 1861 to over 33,000 by 1907 (Neumeier 1995).

## 3 Identification

Identification was possible due to the preserved grave registry for the former cemetery extension, which lists burial location, name, burial date, age at death, and occupational information. Historical records generally classified individuals as male or female. Throughout this article, female and male categories refer to archival classifications unless osteological sex estimation is explicitly discussed.

Following the reconstruction of the original cemetery layout and grave numbering system, entries from the registry were linked to excavated burial locations. Identification was based on the integration of archival, archaeological, stratigraphic, and osteological evidence.

Skeletal age and sex assessments were used as consistency criteria within this process and evaluated together with burial position, stratigraphic relationships, family associations, and other contextual information. Adult age discrepancies were tolerated because of the broader uncertainty associated with osteological age estimation, whereas sex discrepancies were not accepted. For non‐adults, age discrepancies of ±2 years were considered acceptable, while sex was not used as an identification criterion.

Disarticulated remains recovered from grave backfill deposits were inventoried separately and recombined into individuals when coloration, texture, joint surfaces, and age indicators were consistent. These reconstructed individuals were then cross‐referenced with the grave registry and stratigraphic context to evaluate plausibility.

Identification certainty varied according to the quality and consistency of the available evidence. Reduced confidence was primarily associated with reconstruction from grave backfill deposits, stratigraphic disturbances, difficulties in recognizing infant burials during excavation, and occasional inconsistencies in excavation documentation. In several cases, archival records indicated additional non‐adult burials for which no corresponding in situ remains were recovered, although matching skeletal remains were identified among the backfill material. This pattern most likely reflects excavation bias, as shallow infant burials are particularly susceptible to being overlooked during excavation.

Individuals were classified as positively identified when osteological, archaeological, stratigraphic, and archival information converged without contradiction. Cases involving a single remaining source of uncertainty were classified as probable identifications, whereas individuals affected by multiple independent uncertainty factors were excluded from further analysis. A total of 245 individuals were identified. Of these, 142 (58%) were classified as positively identified, while 103 (42%) were assigned a probable identification.

Biographical profiles were compiled using archival sources (see Supporting Information III). Osteological analysis follows the standardized recording protocol of the State Collection for Anthropology Munich (SAM) (Harbeck and von Heyking 2023).

## 4 Composition of the Collection

Individuals in the collection were born between 1806 and 1902 and buried between 1835 and 1910, with most deaths occurring in the 1890s (Figure S3). The archival male/female distribution comprises 136 females (55%) and 109 males (45%), with comparable mean ages at death (females: 34 years; males: 33 years).

**FIGURE 1:**
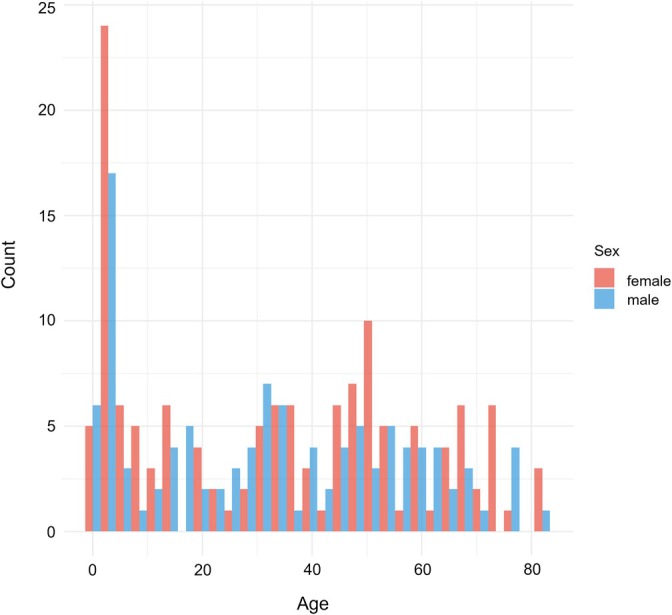
Age distribution by archival male/female classification. For non‐adults, the categories “male” and “female” are based solely on archival records and do not represent osteological sex assessments. Bars represent the number of individuals who died at this age (in years).

The age structure reflects the excavation of the cemetery's designated non‐adult section and includes a substantial proportion of childhood deaths (Figure 1). In total, the collection comprises 90 non‐adults, including 72 individuals under 12 years of age and 18 juveniles.

Most individuals were Catholic (89%), while 23 (10%) were Protestant and 4 (2%) belonged to other or unclear denominations, corresponding to the confessional composition of Neuhausen during this period.

Among adults (≥ 20 years), 83 (57%) were married at the time of death, 45 (31%) unmarried, and 17 (12%) widowed. For individuals recorded as female in the archival sources and aged ≥ 13 years (*n* = 88), the minimum documented number of births ranged from 0 to 14, with a mean of 4 births per individual. These values represent minimum estimates due to potential incompleteness of historical documentation.

Kinship relations were documented for 38 individuals (16%), including parent–child and sibling relationships as well as marital and affinal connections (see Supporting Information IV). The majority of individuals, however, represent unrelated members of a growing urban population.

**FIGURE 2:**
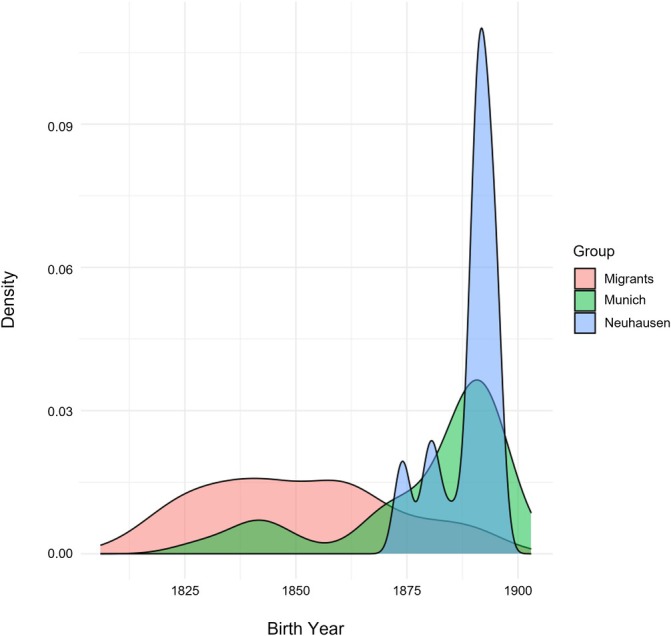
Density plot of birth year distribution, with individuals grouped by place of birth (migrants = born outside of Neuhausen and Munich).

Birthplace is known for 228 individuals (93%). Of these, 29 (13%) were born in Neuhausen or Munich, whereas 199 (87%) originated from rural communities and small towns elsewhere in Bavaria (Figure S4). A smaller proportion derived from other German regions or neighboring countries. Nonmigrants (individuals born in Munich or Neuhausen) were predominantly young non‐adults and adolescents, whereas migrants showed a substantially broader age range and died at older ages on average (Figure 2; see Supporting Information IV for detailed values).

In practice, the adult population of the collection consists almost entirely of individuals born outside Munich and Neuhausen. Nonmigrants cluster in later birth cohorts and are largely represented by those who died at young ages. Migrants, by contrast, were generally born earlier in the 19th century and reached adulthood before relocating to Neuhausen. This pattern corresponds to documented rural‐to‐urban migration processes of the period.

**FIGURE 3:**
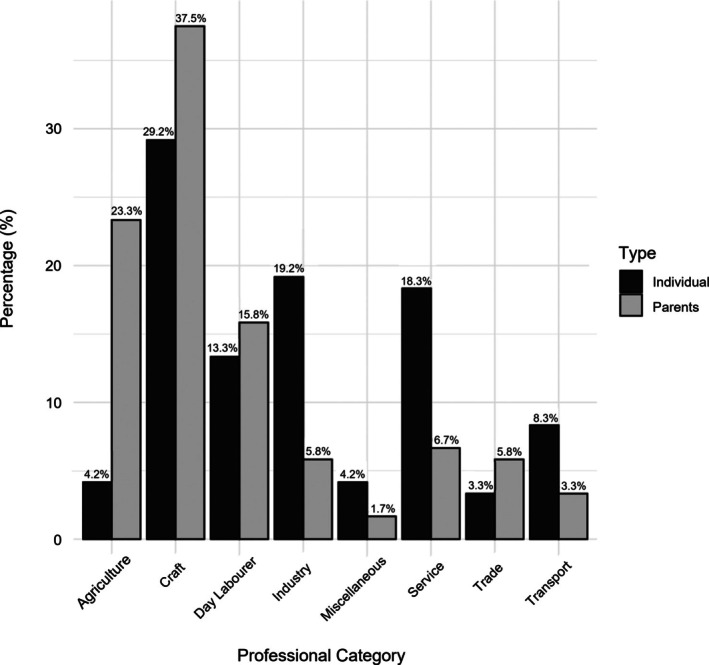
Frequencies of occupational classes among individuals buried at the Winthir cemetery and their fathers (parents). Because non‐adults usually reported only their father's occupation, they were excluded from this figure.

Occupational information is available for 205 individuals (84%), based on the last documented occupation of males, the husband's occupation for married women, and the father's occupation for non‐adults. Occupations were classified into functional categories (agriculture, craft, trade, industry, transport, services, and miscellaneous) to facilitate comparison (Figure 3). These categories were developed for population‐level comparison in the present article; detailed criteria and limitations of the classification are provided in Supporting Information IV.

Most individuals were engaged in manual, craft, or industrial labor, while higher academic or elite professions are largely absent. The craft sector represents the most frequent occupational category (*n* = 63; 31%), followed by service (*n* = 42; 21%), industrial (*n* = 36; 18%), and transport occupations (*n* = 25; 12%).

Parental occupations (*n* = 176) indicate intergenerational differences (Figure 3). Agriculture accounts for 38 cases (22%) among the parental generation but declines to 10% in the deceased generation. Conversely, craft, industrial, and service occupations increase in relative frequency, corresponding to broader economic changes during industrialization.

Burial class can be reconstructed for 238 individuals (97%). Burial classes were administrative categories within the municipal burial system and were largely determined by burial fees and associated grave privileges. They therefore provide a broad indicator of socioeconomic circumstances, although they do not directly reflect individual wealth. The majority were assigned to lower burial categories (Class IV–V), indicating modest socioeconomic conditions (see Supporting Information IV and Figure S6 for details). At the same time, the presence of some higher burial classes and stable public‐sector occupations indicates limited internal socioeconomic differentiation.

A documented cause of death is available for 222 individuals (91%). Historical causes of death were assigned to broad ICD‐11 categories to allow standardized population‐level comparison. The largest category comprises certain infectious or parasitic diseases (*n* = 74; 33%), followed by diseases of the respiratory system (*n* = 36; 16%), circulatory system (*n* = 25; 11%), digestive system (*n* = 18; 8%), neoplasms (*n* = 14; 6%), and external causes (*n* = 13; 6%) (see Supporting Information IV and Figure S7 for details). Deaths attributed to injury, poisoning, or other external causes show a marked male predominance (85%).

**FIGURE 4:**
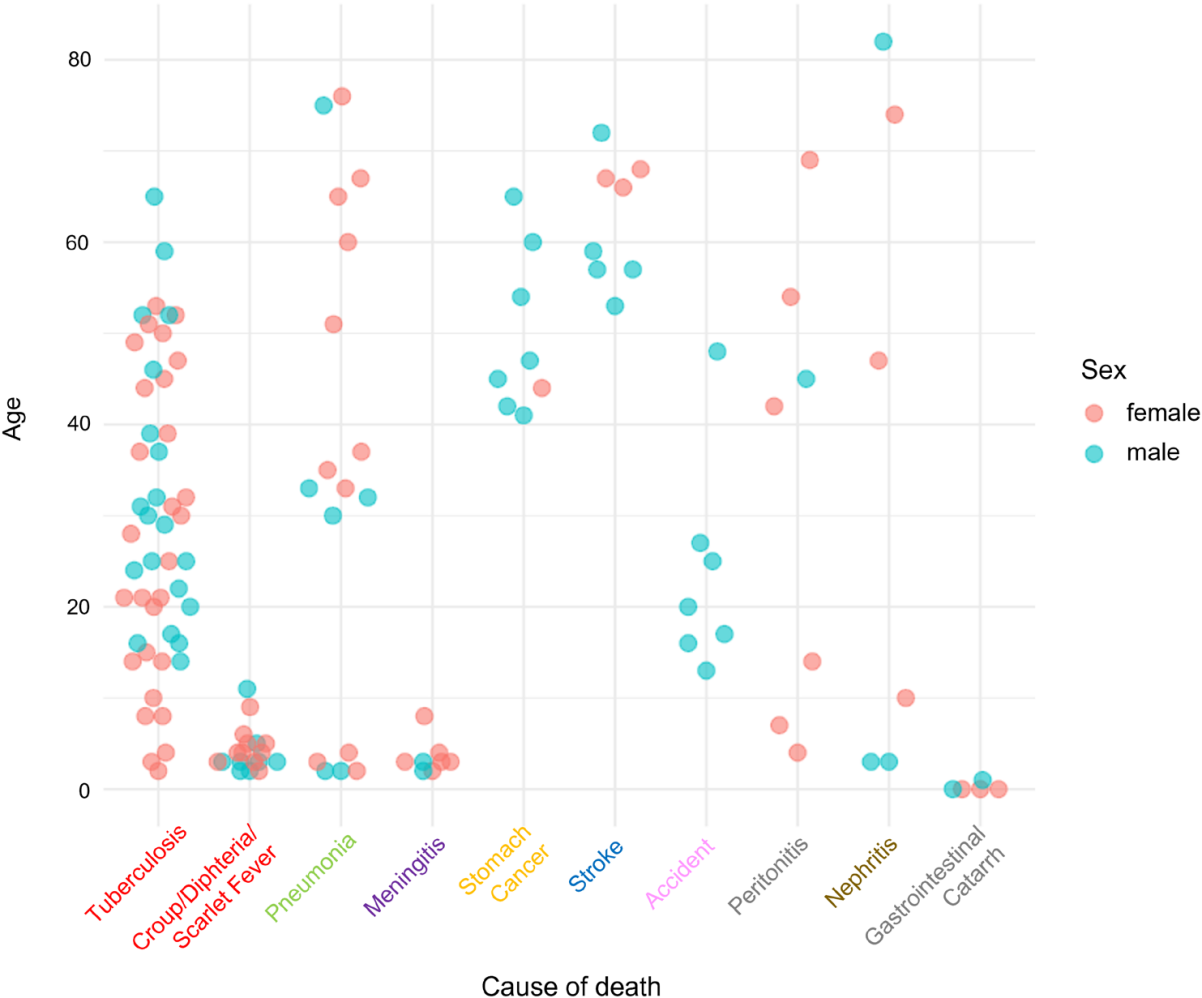
Top 10 causes of death, with x‐axis labels colored according to ICD‐11 categories (red = certain infectious or parasitic diseases, green = diseases of the respiratory system, purple = diseases of the nervous system, orange = neoplasms, blue = diseases of the circulatory system, pink = injury, poisoning or certain other consequences of external causes, gray = diseases of the digestive system, brown = diseases of the urinary system).

Tuberculosis represents the most frequent specific cause of adult death (Figure 4). Among non‐adults, diphtheria, croup, scarlet fever, and gastrointestinal diseases are commonly recorded. Deaths attributed to stroke and stomach cancer occurred primarily in older age at death groups, whereas tuberculosis, peritonitis, and nephritis were recorded across a broad age‐at‐death range (Figure 4).

For approximately 14% of individuals, death occurred in a hospital setting, most commonly at the nearby Red Cross hospital. Within this subgroup, certain infectious or parasitic diseases were less frequent causes of death than in the overall population, whereas neoplasms accounted for a higher proportion.

Overall, the distribution of causes of death corresponds to documented mortality patterns for late‐19th‐century Bavaria, where infectious diseases remained predominant.

## 5 The Skeletal Remains: Preservation and Documented Osteological Features

Alongside the reconstructed biographical information, the Winthir Collection presents a systematically evaluated preservation profile and documented osteological features, summarized here at the population level. Detailed criteria for completeness, surface preservation, fragmentation, dental preservation, and trauma recording are provided in the Supporting Information III.

**FIGURE 5:**
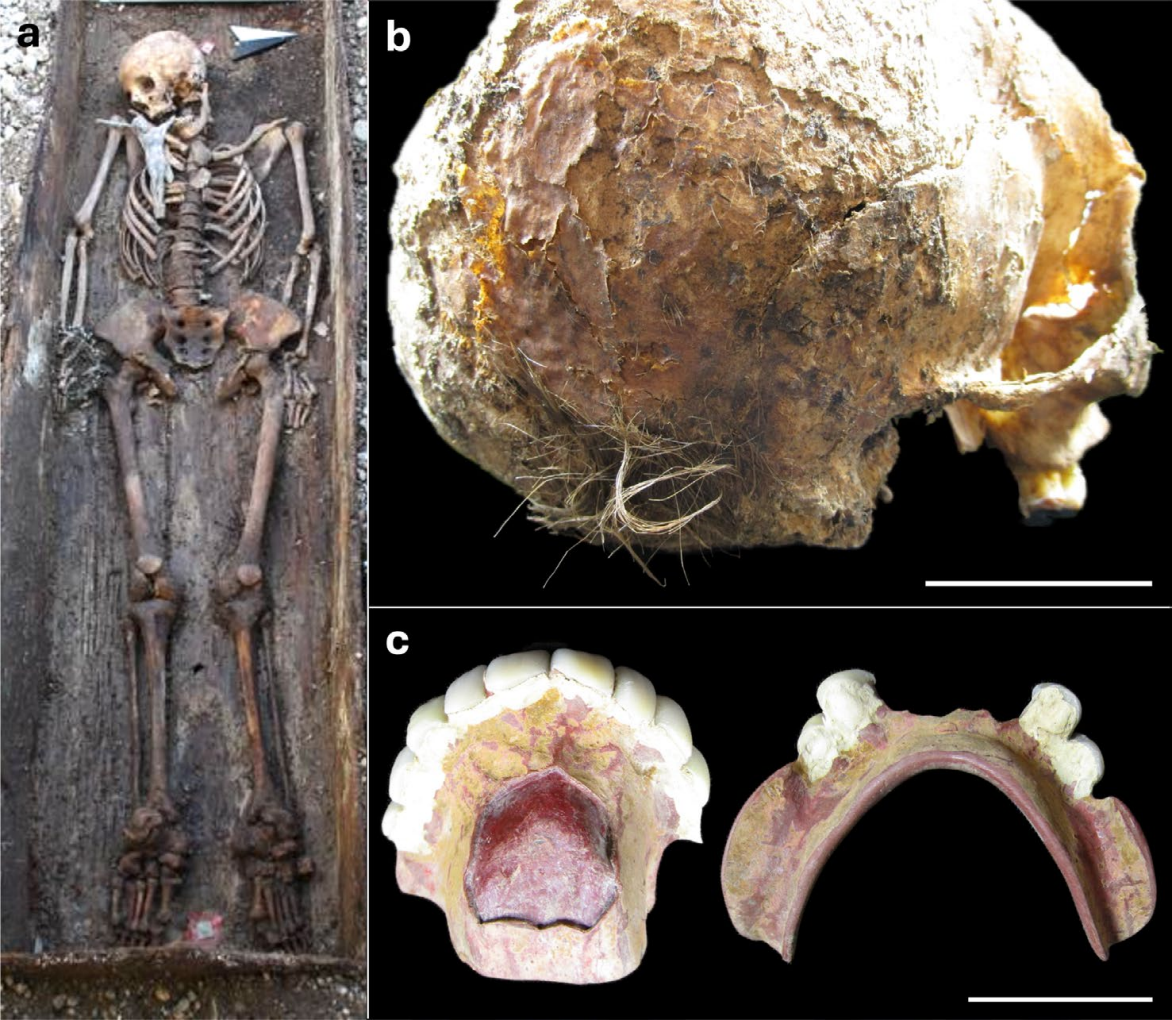
Excellent preservation of the skeletal remains of individual 213 from the Winthir Collection (female; date of birth: May 4, 1858; date of death: October 21, 1895; age at death: 37 years). (a) In situ position; (b) small remnants of soft tissue and hair preserved at the back of the skull (scale = 5 cm); (c) two dental prostheses of the individual (scale = 3 cm). She was buried in the Winthir Cemetery with burial category IV. The cause of death is listed as heart failure after pneumonia with pulmonary embolism. She lived in Munich since 1886 and was originally from Salzburg. She was Catholic, unmarried, no children are documented, and worked as a cook and cashier. As a side business, she traded in delicatessen and food. Her father was a small farmer and mason.

Completeness was most frequently scored as excellent (57%; Figure 5), followed by good (28%), fair (9%), and poor (7%). Complete absence of a skeletal region was most frequent for the pelvis (9%), followed by the skull (7%) and the shoulder girdle (6%). Most individuals (85%) showed no completely missing major region. The most common absence patterns involved either a single missing region or paired absences involving the pelvis.

Surface preservation was most often characterized by moderate damage (47%) or none to minor damage (42%), whereas severe damage occurred in 12% of individuals. Fragmentation showed a comparable distribution: moderate fragmentation predominated (47%), closely followed by none to minor fragmentation (46%), while severe fragmentation was rare (7%).

Additional mineralized tissues were observed in 11 individuals (see Supporting Information V for illustrations and contextual examples). Unfortunately, the location of these tissues was documented in only a few cases during excavation. Where localization was possible, the tissues originated from the abdominal cavity (*n* = 1), the neck and chest area (*n* = 1), or the pelvic region (*n* = 1), and were described as pleural calcification in one case. These findings encompass a range of heterotopic ossifications or pathological calcifications associated with trauma, surgery, infection, inflammation, or other processes (Assis and Garcia 2023; De Luca and Del Piero 2026; Hwang et al. 2018) and may offer potential for further study (Jerković et al. 2018).

Dental preservation was quantified using two complementary indices (see Supporting Information III for details). First, the percentage of postmortem tooth loss in the full sample (*n* = 245) shows a strongly right‐skewed distribution (mean = 24%; median = 6%). A total of 105 individuals (43%) exhibit no postmortem tooth loss, whereas 23 individuals (9%) show complete postmortem tooth loss. Second, the percentage of remaining teeth was calculated for adults only (*n* = 175). The mean percentage of remaining teeth was 45% (range: 0%–100%). Tooth retention was distributed as follows: 16% (*n* = 28) at 0%, 18% (*n* = 31) at 0%–25%, 22% (*n* = 39) at 25%–50%, 21% (*n* = 36) at 50%–75%, 21% (*n* = 36) at 75% to < 100%, and 3% (*n* = 5) with complete retention. Dental calculus was present in 146 individuals (60%). Metal dental fillings were identified in three individuals, and two individuals possessed dental prostheses (Figure 5).

To illustrate the diversity of osteological information represented within the collection, selected observations from adult and non‐adult individuals are briefly summarized here: Tibial squatting facets were observed in 27% of assessed adults, while cribra orbitalia occurred in 28% of adults and 61% of assessable non‐adult crania. Endocranial new bone formation was recorded in 30% of assessed non‐adults. More detailed evaluations of these markers are available elsewhere (Harbeck and Paulus in press).

Skeletal trauma and evidence of craniotomy represent additional categories of osteological information that can be evaluated at the population level within the collection. Evidence of pre‐ or perimortem trauma was observed in 41 of 245 individuals (17%). Trauma was more frequently documented among individuals classified as males (24%) than among those classified as females (11%). The majority of cases represent healed antemortem injuries, affecting 39 individuals (16%). In 11 individuals, trauma involved multiple anatomical regions. Across all pre‐mortem cases, 57 skeletal elements with visible healed trauma were recorded, most frequently affecting ribs (30%), followed by the radius (14%) and hand or foot bones (12%). Clear perimortem trauma was documented in two individuals. In one adult female, cranial alterations correspond to surgical intervention shortly before death. In one adult male, multiple unhealed fractures affecting upper and lower limbs, ribs, and vertebral processes correspond to archival documentation of a fatal railway accident (for more details on these individual cases see Harbeck and Paulus in press).

Skeletal signs of craniotomy were observed in 10 of 218 assessed individuals (5%; see Figure S13 for example). Archival research indicates that autopsy was performed in nine cases. However, skeletal evidence of craniotomy and archival documentation of autopsy co‐occur in only four individuals; six show skeletal evidence alone, and five have only archival documentation indicating autopsy. Where skeletal evidence is present without documentation, it may reflect undocumented autopsy or anatomical dissection, consistent with contemporary medical practice (Gulczyński et al. 2010).

## 6 Research Potential, Access, and Curation

The Winthir Collection is a documented historical skeletal collection from late‐19th‐century southern Germany in which osteological remains are linked to reconstructed biographical data. The integration of demographic, occupational, and migration data enables investigation of urbanization processes and mortality patterns during industrialization.

The collection also provides a basis for methodological work in biological anthropology. Especially the comparatively large and identified non‐adult component broadens the regional reference sample for studies of growth, development, age estimation, and childhood morbidity. This is particularly relevant because identified historical skeletal collections in Central Europe contain only very small numbers of non‐adult individuals. At present, approximately 20 identified non‐adult skeletons from nonmodern Central European series are documented (see Supporting Information I). The Winthir Collection therefore increases the number of identified non‐adult remains available for research in this region and contributes to closing a noticeable gap in comparative material.

Beyond its age structure, the assemblage reflects a generational contrast between predominantly rural‐born adult migrants and locally born children of migrant parents. This configuration offers a defined context in which questions of early‐life environment, migration, and urban transformation can be examined in relation to skeletal findings.

Skeletal evidence of medical interventions and postmortem cranial intervention, together with archival indications of cause of death or that autopsy was performed in some cases, permits comparison between osteological observations and documentary sources. Detailed medical records were not systematically examined yet, and it remains unclear to what extent such documentation has been preserved. Further archival work may clarify this issue.

The collection is curated at the SAM. The remains derive from a documented Christian cemetery context and are not associated with unjust acquisition. Ethical admissibility is assessed in accordance with established German museum and professional guidelines (DMB 2013; Grupe et al. 2015; Preuß 2007). Within these frameworks, scientific interest in preservation and research is weighed against the interests and norms of people living today, taking into account the provenance and acquisition context of the remains.

Given the possibility of individual identification, postmortem personal rights and potential interests of living descendants are considered. Archival protection periods are respected, surnames are omitted from publicly accessible information, historical research is limited to the lifetime of each individual, and no genealogical tracing is undertaken.

Osteological, historical, and contextual information is recorded in a standardized format and made available through a structured open‐access catalog (Harbeck and Paulus in press). The catalog documents completeness and notable skeletal features as well as the available archival information, including identification certainty and preservation status.

The skeletal material is available for nondestructive scientific research upon request and in accordance with institutional and ethical regulations.

## Author Contributions

**Kristin von Heyking:** investigation, writing – review and editing, formal analysis, data curation. **Michaela Harbeck:** supervision, data curation, project administration, writing – review and editing, visualization, methodology, writing – original draft, funding acquisition, conceptualization. **Albert Zink:** writing – review and editing, funding acquisition, resources. **Elsa Seyr:** investigation, writing – review and editing, validation. **Jochen Haberstroh:** conceptualization, writing – review and editing, data curation. **Maren Velte:** validation, writing – review and editing, investigation. **Barbara Schratzenstaller:** investigation, writing – original draft, writing – review and editing, data curation. **Eva Kropf:** writing – review and editing, investigation. **Bernd Trautmann:** investigation, data curation, writing – review and editing. **Ja Young Lee:** investigation, writing – original draft, visualization, writing – review and editing, data curation. **Christof Paulus:** conceptualization, investigation, funding acquisition, writing – original draft, methodology, project administration, supervision.

## Funding

This work was supported by Deutsche Forschungsgemeinschaft (426344714).

## Supporting information


**Data S1:** Supporting Information.
**Figure S1:** Documented osteological collections in Central Europe.
**Figure S2:** Overview of the excavation plan of the old section of the Winthir Cemetery, created with QGIS 3.22 and based on Stremke et al. (in press).
**Figure S3:** Number of births and deaths by year.
**Figure S4:** Places of origin of the individuals buried in the Winthir Cemetery in Neuhausen (purple = men, red = women), map created using Q‐Gis (version 3.34.11‐Prizren) and the European Digital Elevation Model (DGM1000 (GeoBasis‐DE/BKG [2024])).
**Figure S5:** Distribution of occupational classes.
**Figure S6:** Number of individuals per adult burial category by sex (Armenklasse = Pauper's burial).
**Figure S7:** Frequency of ICD‐11 disease category.
**Figure S8:** Undefined, mineralized tissue with unknown in situ position from individual 82 (female: 65 years). Scale: 2 cm.
**Figure S9:** Undefined, mineralized tissue with unknown in situ position from individual 89 (female: 51 years). Scale: 2 cm (left) and 3 cm (right).
**Figure S10:** Undefined, mineralized tissue found at the chest and throat region of individual 98 (female: 70 years). Scale: 1 cm.
**Figure S11:** Mineralized tissue with unknown in situ position of individual 121 (male: 52 years). Scale: 4 cm.
**Figure S12:** Mineralized tissue found in the pelvis region of individual 150 (female: 46 years). Scale: 2 cm.
**Figure S13:** Skull of individual 114 (female: 44 years) with signs of a postmortem autopsy. Scale: 5 cm.

## Data Availability

The data supporting the findings of this study are contained within the article, its Supporting Information, and the published catalogue of the Winthir Collection (Harbeck & Paulus, 2026). The analyses presented are based on information compiled from these sources.
