# Supplementary material for: The Winthir Collection: An Identified Historical Skeletal Series From Munich, Germany
Source: Am J Biol Anthropol. 2026 Jun 25;190(2):e70302. doi: 10.1002/ajpa.70302 (PMC13303109; doi:10.1002/ajpa.70302)
Supplement: Supplementary file 1 — Data S1: Supporting Information. Figure S1: Documented osteological collections in Central Europe. Figure S2: Overview of the excavation plan of the old section of the Winthir Cemetery, created with QGIS 3.22 and based on Stremke et al. (in press). Figure S3: Number of births and deaths by year. Figure S4: Places of origin of the individuals buried in the Winthir Cemetery in Neuhausen (purple = men, red = women), map created using Q‐Gis (version 3.34.11‐Prizren) and the European Digital Elevation Model (DGM1000 (GeoBasis‐DE/BKG [2024])). Figure S5: Distribution of occupational classes. Figure S6: Number of individuals per adult burial category by sex (Armenklasse = Pauper's burial). Figure S7: Frequency of ICD‐11 disease category. Figure S8: Undefined, mineralized tissue with unknown in situ position from individual 82 (female: 65 years). Scale: 2 cm. Figure S9: Undefined, mineralized tissue with unknown in situ position from individual 89 (female: 51 years). Scale: 2 cm (left) and 3 cm (right). Figure S10: Undefined, mineralized tissue found at the chest and throat region of individual 98 (female: 70 years). Scale: 1 cm. Figure S11: Mineralized tissue with unknown in situ position of individual 121 (male: 52 years). Scale: 4 cm. Figure S12: Mineralized tissue found in the pelvis region of individual 150 (female: 46 years). Scale: 2 cm. Figure S13: Skull of individual 114 (female: 44 years) with signs of a postmortem autopsy. Scale: 5 cm. [file AJPA-190-e70302-s001.docx]

**Supplementary**

**I Documented osteological collections in Europe**

Efforts to globally map identified collections have recently been advanced by Petaros et al. (2021), who provide an interactive map on the Forensic Anthropology Society of Europe’s (FASE) website. This map suggests the presence of several identified skeletal collections in Central Europe, though closer examination reduces the number (Fig. S1): A quarter of the FASE-listed collections consist of either entirely or partially of single bone elements, anatomical specimens, or unspecified skeletal materials, which may serve as reference collections only for highly specific research questions (Petaros et al. 2021). These include disease-specific specimens (e.g., the Galler Collection; Rühli et al. 2003), which are not shown in Fig. S1, and skull collections—shown as blue circles in Fig. S1—such as the Virchow Collection in Berlin, primarily comprising unidentified skulls collected from various countries in the late 19th and early 20th centuries (Heeb & Teßmann 2021; Stoecker et al. 2013). Notably, for many collections, we could not find any information on the websites of their stated home institutions, nor citations in recent literature (as indicated by a Google Scholar search from 1990 to present). This lack of information may suggest that many of these are not either longer in active use or accessible. It is also possible that these entries in the phase map are only single individuals or archaeological skeletal series that contain no or only isolated identified individuals. Already Petaros et al. (2021) noted similar limitations, observing that data for about a quarter of the mapped collections were either unavailable or incomplete.


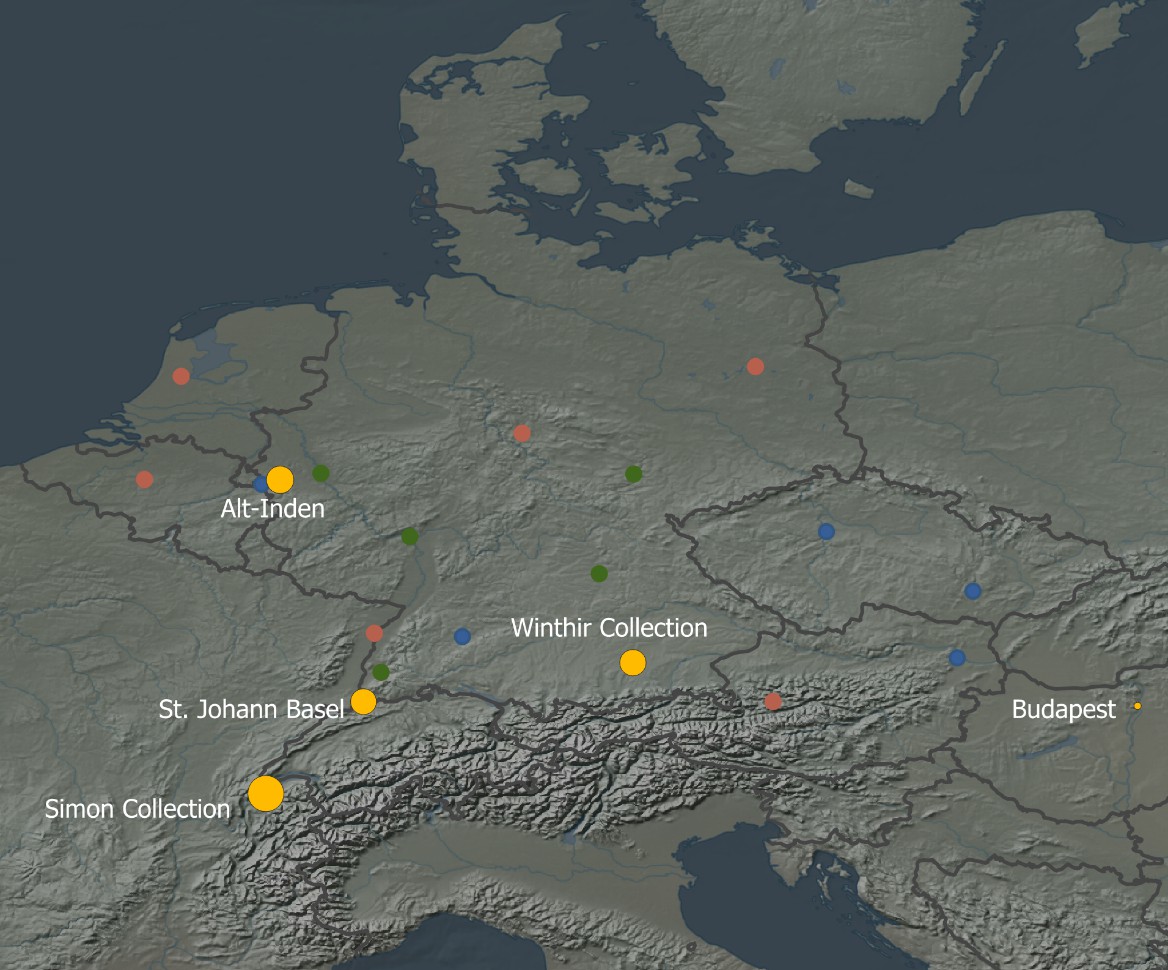


Fig. S1. Documented osteological collections in Central Europe. Yellow = non-modern identified osteological collections (size according to number of individuals); blue = modern anatomical collection from body donations; red = skull collections; green = collections that appear on the FASE map of identified osteological collections, but for which no identified individuals could be verified (map data produced using Natural Earth free vector and raster map data, illustration: QGIS 3.34). Based on information from the Map of Identified Osteological Collections. Forensic Anthropology Society of Europe (FASE; https://www.google.com/maps/d/edit?mid=162_ElRDZuDCJfM10jCkPpRSFPSw&ll=31.412645801177902%2C0&z=2).

Historical identified collection includes a small collection of 10 identified individuals listed for Budapest. That may correspond to the non-mummified remains from the Dominican Church in Vác, identifiable by coffin inscriptions, as well as a lesser-known, identified skeleton collection from Germany, which is housed at the Georg-August-University in Göttingen (Szikossy et al. 1997). It consists of 236 individuals buried between 1877 and 1924 in Alt-Inden, North Rhine-Westphalia, with Church records providing names, age at death, date of burial, and in some cases, cause of death and occupation (Salega & Grosskopf 2022).

Furthermore, two of the most comprehensive identified non-modern collections in Central Europe are in Switzerland. The St. Johann Collection (also called Basel-Spitalfriedhof) derived from a hospital cemetery in Basel that was used from 1845 to 1868. Over 1000 skeletons have been excavated there, the majority of which have been identified, and medical records are available for most of them (Hotz et al. 2016). But unfortunately, most of the skeletal remains were reburied after excavation and only the remains of a maximum of 270 identified individuals appear to be preserved as complete skeletons and accessible in the Natural History Museum in Basel (Aebi et al. 1989; Allemann et al. 2016).

The Laboratory of Prehistoric Archaeology and Anthropology of the University of Geneva is housing the SIMON Identified Skeletal Collection (also called the Gemmerich Collection), containing 496 Swiss individuals (e.g., Abegg et al. 2023). It originates from late 19th-mid-20th-century tombs from cemeteries and biographic data of the individuals are known through tombstones and official death registers (Indra et al. 2021). The SIMON Collection includes 14 subadults, mostly older adolescents (Abegg et al. 2023; Indra et al. 2021). The Alt-Inden Collection comprises approximately five children or adolescents (Salega & Großkopf 2022). For the St. Johann Collection, no individuals under 17 years of age have been documented (Usher 2002).

**II Cemetery structure**

Fig. S2. Overview of the excavation plan of the old section of the Winthir Cemetery, created with QGIS 3.22 and based on Stremke et al. (in press). The cemetery is divided into sections 9 and 10, separated by pathways. Within each section, burial plots are arranged in rows (dark grey: 2014 excavation; light grey: 2018 excavation). A portion of Section 10 is characterized by notably smaller burial pits containing only young children. The former cemetery was enclosed by two perimeter walls, and graves aligned along these walls show individuals oriented with their heads placed against the wall (Section ML; “left Wall row” in the figure). The excavation also identified several disturbances caused by recent soil interventions, some of which have obliterated entire areas of the cemetery (grey-dotted zones).


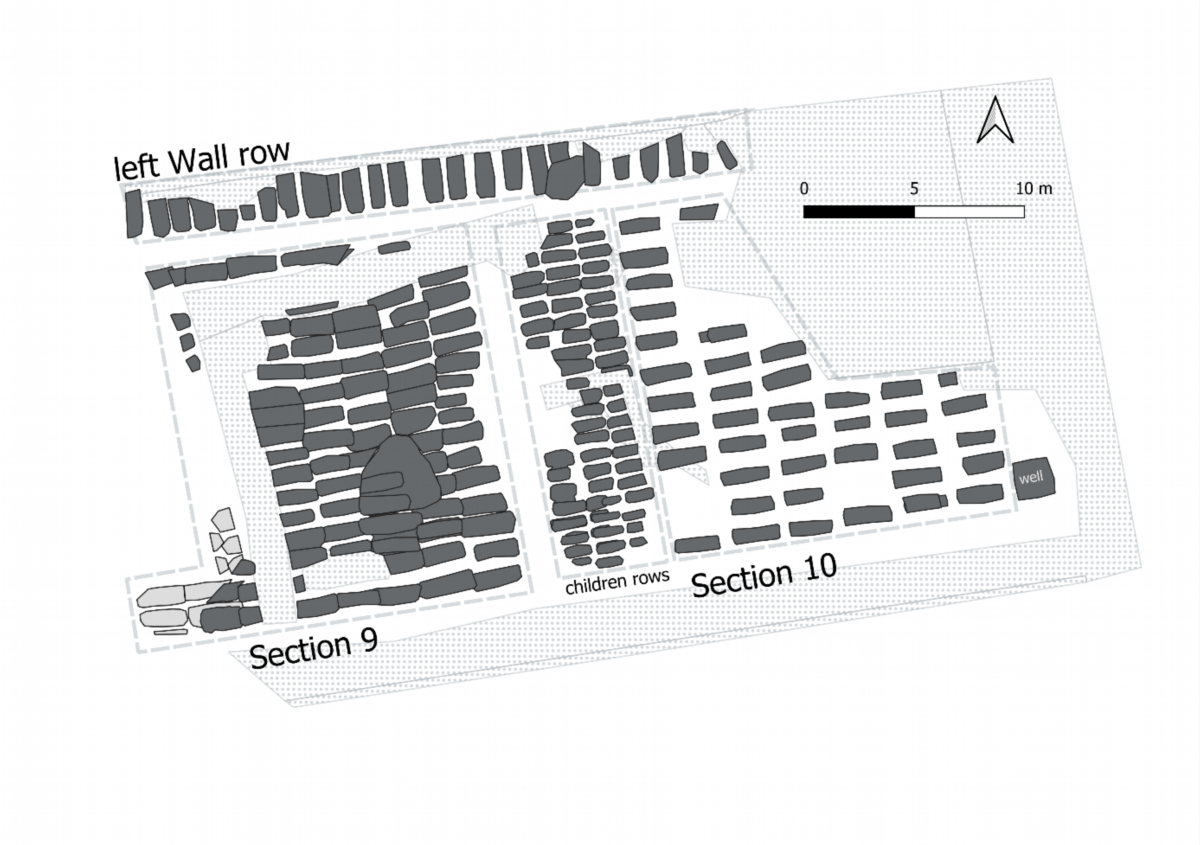


**III Detailed Method information**

***Historical research***

Using the grave registry as a starting point, each burial was systematically linked to additional archival sources in order to verify identities and expand biographical profiles. Church and municipal death registers of Neuhausen and, after 1890, the city of Munich provide information on dates and causes of death, religious affiliation, marital status, parental names, and birthplaces. Police registration records of Munich (Stadtarchiv München), documenting all persons residing or working in the city, were particularly informative. These registration sheets typically list household members, occupations, migration histories, addresses, and changes in family structure over time. Married women and children commonly appear under the husband’s or father’s registration, which allows reconstruction of household networks. When individuals had resided outside Munich for extended periods, additional municipal and regional archives were consulted to obtain complementary birth, marriage, and occupational data, particularly for those originating from rural Bavaria or neighboring regions. Governmental sources were supplemented, where available, by church baptismal and marriage records, newspaper reports, and personnel files from larger institutions (e.g., the Munich Postal Service Headquarters, the Roads and Waterways Department, and military archives).

The biographical profile systematically reconstructed for each individual includes place of birth, marital status, date of birth, religious denomination, age at death, date of death, and cause of death (as listed in the death registers). In addition, the following information was collected:

- **Burial category**: Descendants could choose among five burial classes (three for children), including—since 1890—a pauper’s funeral option for indigent individuals. These classes differed in features such as the hearse, number of clergy, casket type, clothing of the deceased, decorations, and floral arrangements.
- **Minimum number of births for women**: Police registration records were used as the primary source, as they list a family’s or single woman’s children with dates and places of birth, including children born before moving to Munich. Because only children still in childhood and not yet fully employed were recorded, church baptismal registers were additionally examined for the women’s known places of residence for ages fifteen to forty-five, using maiden names and, after marriage, married names. Municipal registration records from locations outside Munich were also consulted when possible. Despite these efforts, the number of births must be regarded as a minimum, since not all births or places of residence during the full reproductive span could be identified.
- **Age of the mother at birth**: The mother’s age at the birth of each child was recorded.
- **Occupation**: For men and unmarried women, all recorded occupations were listed; for married men and women, the occupations of their spouses were added. For married, divorced, or widowed women, occupations held before marriage or after the husband’s death were also documented. For children, compulsory school attendance was only recorded when explicitly designated as “schoolchild” in a source.
- **Occupation of the parents**: The occupations of the parents were documented for all individuals.
- **Further information:** Whenever available, additional medical or contextual information was recorded. This includes references to medical procedures such as autopsies documented in the death registers, as well as any evidence of familial relationships derived from police registration records, church registers, or other archival sources. Moreover, when newspaper reports provided more detailed descriptions of the circumstances or causes of death, these data were incorporated to refine and contextualize the recorded cause of death.

***Osteological examination***

A full inventory was recorded for each individual. Age estimation for adults employed the pubic symphysis, auricular surface, and, when necessary, cranial suture closure (Buckberry & Chamberlain 2002; Herrmann et al. 1990; Nemeskéri et al. 1960). Sex estimation relied on pelvic and cranial morphology (Harbeck & von Heyking 2023). Subadult age estimation was based on dental eruption and skeletal maturation indicators (AlQahtani et al. 2010; Baker et al. 2005; Schaefer et al. 2009; Scheuer & Black 2000; Ubelaker 1978). The SAM-standard documentation also includes the recording of preservation status, selected osteological traits, and metrics (Harbeck & von Heyking 2023). A preliminary overview of these features is given in Harbeck & Paulus (in press). Here, we examine the preservation of the remains in more detail and give an overview of evident pre- and perimortem trauma in relation to the historical record. For this purpose, preservation status and traumatic alterations were evaluated for each anatomical region (skull, vertebral column and ribs, pelvic girdle, shoulder girdle, upper limbs, lower limbs) according to SAM protocols. A region was considered assessable when at least one element of that region was present; it was classified as unobservable when none of the required skeletal elements were available. Consequently, we report frequencies at the anatomical-region level rather than at the level of individual skeletal elements (e.g., femur), acknowledging that variation in element preservation within each region cannot be excluded.

In assessing preservation, we distinguished between completeness, surface preservation, and degree of fragmentation. To evaluate completeness, we developed a composite “completeness score” that integrates both the preservation quality of present anatomical regions and the total absence of others. Each anatomical region (skull, spine, pelvis, shoulder girdle, upper extremities, lower extremities) was first assigned a preservation score: 1 = good preservation (>75% complete), 2 = moderate preservation (25–75% complete), 3 = poor preservation (<25% complete), and 0 = completely absent. For each individual, the average preservation of present (i.e., assessable) regions was calculated as follows: if S is the sum of preservation scores of present regions and N is the number of present regions, then the average preservation score is S/N. Missing regions were incorporated through a penalty term: if M is the number of missing regions and f is the penalty factor (set to 0.5), then the penalty equals f × M. The final completeness score is the sum of the average preservation score and the penalty, i.e**., Completeness Score =** S/N **+ (**f × M**).** For interpretability, completeness scores were categorized as follows: excellent ≤ 1.5; good > 1.5–2.5; fair > 2.5–3.5; poor > 3.5. This scoring approach ensures that individuals with a higher number of missing anatomical regions receive proportionally lower completeness assessments. For each individual, overall bone surface preservation and fragmentation were also evaluated using the numerical scheme of Grupe et al. (2015), in which 0 indicates an unassessable or absent region, 1 well-preserved or minimally fragmented bone (>75% preserved), 2 moderately preserved or partially fragmented bone (25–75%), and 3 poorly preserved or highly fragmented bone (<25%) (Harbeck & von Heyking 2023). Additionally, any preserved soft tissue, hair or heterotopic ossification was documented.

With respect to dental preservation, the presence or absence of each jaw section, intravital and postmortem tooth loss, and any resulting loss of entire jaw segments were recorded. These data were used to calculate the percentage of teeth lost per individual, based on a total of 32 teeth (permanent/mixed dentition) or 20 teeth (deciduous dentition). Following the scheme applied to skeletal preservation, dental preservation was categorized as: good (0–25% teeth lost), moderate (25–75%), and poor (>75%). For individuals with permanent dentition, the percentage of remained teeth was also calculated. This value was always equal to or lower than the percentage of teeth lost because antemortem and unerupted teeth were also included in the calculation. Visible calculus deposits, as well as dentures or dental fillings, were also documented.

Signs of trauma are recorded for each body region. Skeletal elements with skeletal trauma were described in detail and examined macroscopically of evidence of healing. If this could be determined the underlying injury was classed as antemortem. If no evidence of healing was present the trauma was classed as perimortem. While the cause of each of the observed premortal trauma cannot be discussed further here, the few cases of perimortem trauma shall be attributed to different causes. Perimortem fractures were distinguished from postmortem damage based on the coloration of the bone at the fracture and fracture morphology. As a special type of perimortem trauma, signs of medical, post-mortem autopsies on the cranium are distinguished from other traumas, whereby the unmistakable incision at the cranium was considered a reliable sign.

**IV Further details on Composition on the Collection**

***Birth and Death years***


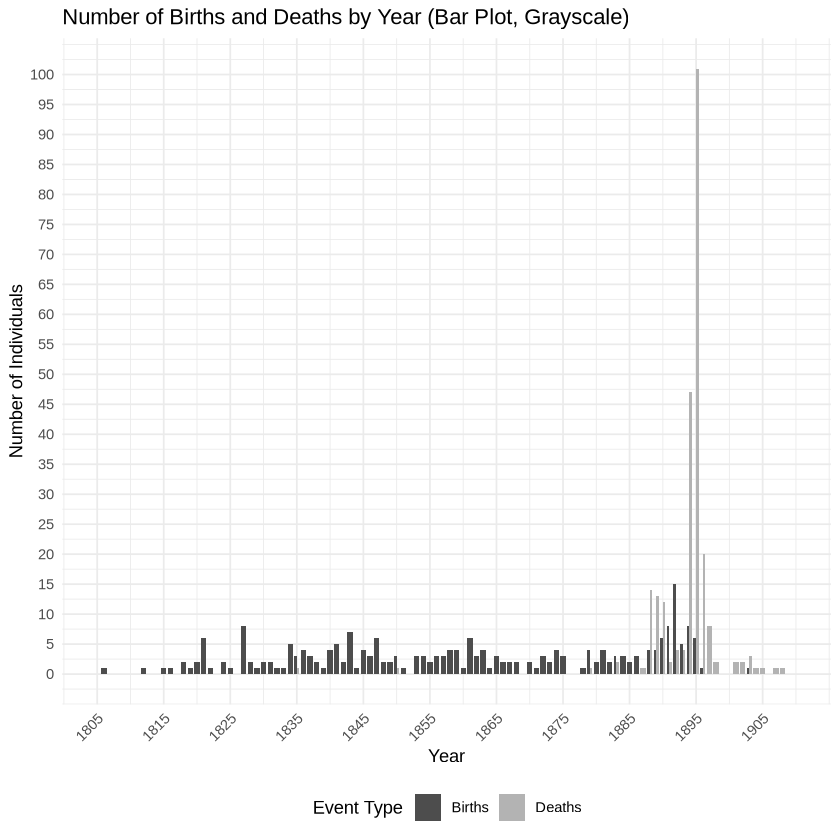


Fig. S3. Number of births and deaths by year. Bars represent the number of individuals who were born (dark grey) or died (light grey) in each year.

***Kinship details***

For some individuals, kinship relationships were determined: First-degree biological relationships were identified for 21 individuals (5 parent–child pairs, 8 sibling pairs, and one set of three siblings), and second-degree relationships for 4 individuals (2 grandparent–grandchild pairs and 1 pair of half-siblings). Only 9 individuals were involved in more than one family relationship, always forming groups of three related individuals: in 2 cases, one parent with two children (full siblings), and in one case, a pair of siblings and their nephew. Fourteen individuals were connected to one another but were not biologically related (4 married couples, 2 parents-in-law/child-in-law constellations, and one step-grandparent relationship).

***Place of origin***

To characterize the nature of individuals’ birthplaces, locations were classified according to population size: hamlet (≤50 inhabitants), village (51–500), small town (501–3,000), medium-sized town (3,000–5,000), large city (5,001–80,000), and metropolis (>80,000). Population figures were taken from the 1953 Historical Municipal Directory, which provides population data for Bavarian municipalities from 1840 to 1952. The population figure for 1875 was used; if unavailable, the nearest earlier or later figure within ±5 years was selected. In rare cases—most often hamlets—no population data were provided. If a locality was classified as a Bavarian hamlet in modern sources, we assumed that this classification also applied historically.

The place of origin could be recorded for 228 out of the 245 individuals. Of them only 33 Individuals were born in Neuhausen (14.5%) another 44 (19.3%) were born in nearby Munich, and 13 were born outside of Bavaria (5.7%). They came from several different places, mostly from Austria (6 individuals) and other places of the Germany Empire (one individual each from Rhineland, Nordrhein-Westfalen, Baden-Württemberg, Thuringia). One individual each came from Poland and Belgium.

However, the majority of the individuals of the Winthir Collection (138 individuals) were born in other regions of Bavaria (60.5%). Beside of Munich, Thierhaupten was named for four individuals, while Augsburg, Landsberg am Lech and Würzburg were named for two individuals each. All other birthplaces in the dataset appeared only once.

The migrants were evenly distributed across Bavarians small towns (26.8%), villages (25.4%), and medium sized towns (22.5%). The remaining migrants were from hamlets (14.5%) or large towns/another metropole region (2%). Fig. S4 shows that the places of origin were scattered for both men and women all over Bavaria. Except for Munich and Neuhausen, it was very rare for more than one individual to come from the same place.


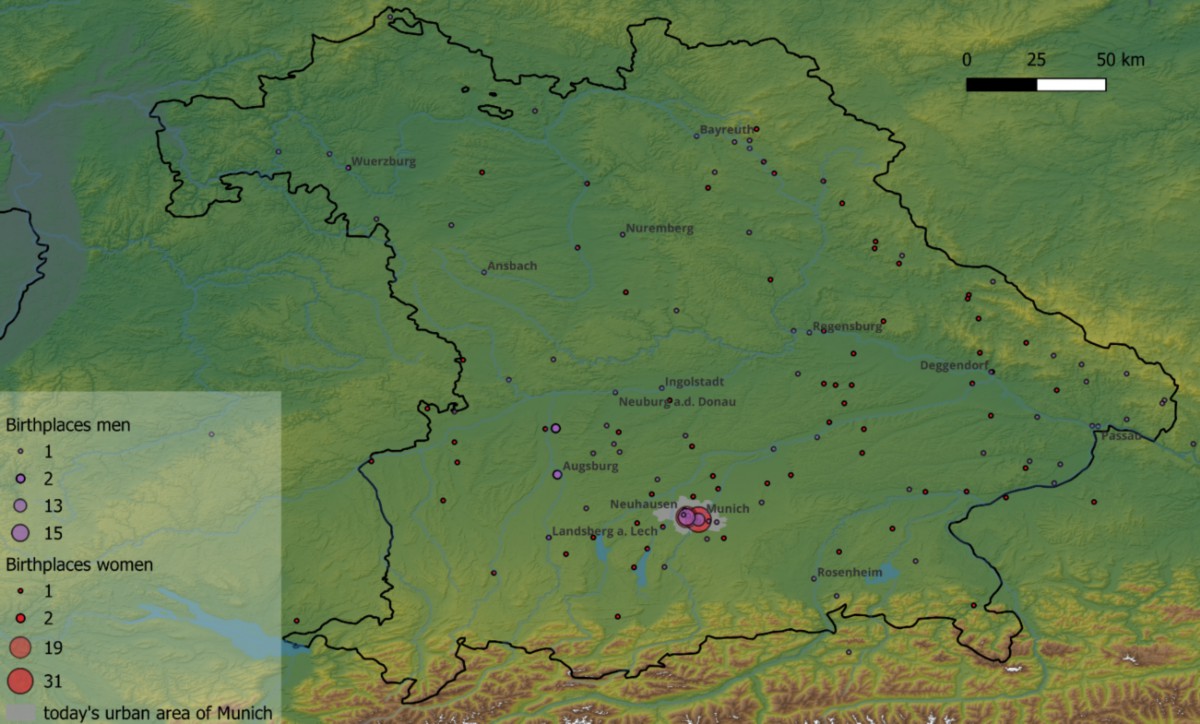


Fig. S4. Places of origin of the individuals buried in the Winthir Cemetery in Neuhausen (purple = men, red = women), map created using Q-Gis (version 3.34.11-Prizren) and the European Digital Elevation Model (DGM1000 (© GeoBasis-DE / BKG (2024)). Marker size indicates the number of men and women originating from that location. Not all places of origin for migrants from outside of Bavaria are shown. Names are given for large city (5,001–80,000 inhabitants) and Metropolis (over 80,000 inhabitants) of the Bavarian Kingdom around 1875.

To better understand demographic differences, individuals were grouped into:

- Non-migrants: born in Neuhausen or Munich
- Migrants: born outside Munich/Neuhausen (including the rest of Bavaria, other German regions, and abroad)

This comparison reveals clear contrasts (Fig. 2): Non-migrants were predominantly children and adolescents, with a mean age at death of 5.7 years (range: 0–20 years, SD: 6). Migrants showed a much wider age range (0–82 years) and died at significantly older ages (mean: 36.7 years, median: 38 years, SD: 23.5). Non-migrants clustered strongly around birth years 1880–1895, while migrants displayed a much broader distribution, having been born throughout the 19th century, with a notable concentration in the 1840s to 1870s. Overall, migrants tended to be older and were born earlier, whereas Munich- and Neuhausen-born individuals were younger and born later, which may reflect a broader demographic divide. No sex differences were found between migrant and non-migrant distributions.

***Occupational and Social Background – details***

To obtain an impression of the social backgrounds of individuals buried in the Winthir Cemetery, the recorded occupations of men were analyzed (Fig. S5). This included not only the occupations of male individuals buried in the cemetery, but also those of fathers (in the case of children) and husbands (in the case of married women). Occupations of unmarried women and of fathers of illegitimate children were excluded. Classifying 19th-century occupations is challenging for several reasons and various systems for occupational classification in this period have been proposed.

Individuals frequently held several occupations during their lifetimes. For the present overview, only the last documented occupation was considered. In cases of simultaneous occupations, the more specific occupation was chosen. For example, when both a craft and a day-laborer occupation were listed, the craft occupation was selected. Occupations not actively practiced alongside the primary trade were not included.

Occupations were classified according to functional categories:

- **Agriculture:** cultivation, livestock management, and forestry (e.g., farmer, farmhand, shepherd)
- **Craft:** manual production and repair of goods, typically structured in master–journeyman–apprentice systems
- **Trade:** purchase and sale of goods (e.g., trader, merchant, innkeeper)
- **Industry:** factory-based or large-scale production associated with industrial manufacturing (e.g., boilermaker, factory worker, spinner, weaver)
- **Transport:** transportation of goods and people (e.g., railway workers, other transport personnel)
- **Services:** provision of personal or public services (e.g., hairdresser, teacher, physician, musician) and occupations serving the state (e.g., civil servant, soldier, police officer, postman, lithographer)
- **Miscellaneous:** categories not fitting the above (e.g., private individuals, pensioners)

We acknowledge the limitations of this system. Industrialization blurred the boundaries between crafts and industry, as artisanal work was increasingly carried out in factory settings. A boilermaker, for instance, could work either in a small workshop or in an industrial plant. In this study, boilermakers were classified as industrial workers because workplace information consistently indicated industrial employment. In contrast, trades that remain recognized as crafts today (e.g., stonemasons), and are still associated with guild-based training systems, were classified as crafts—even when workplace statements suggested that they sometimes operated in industrial contexts.


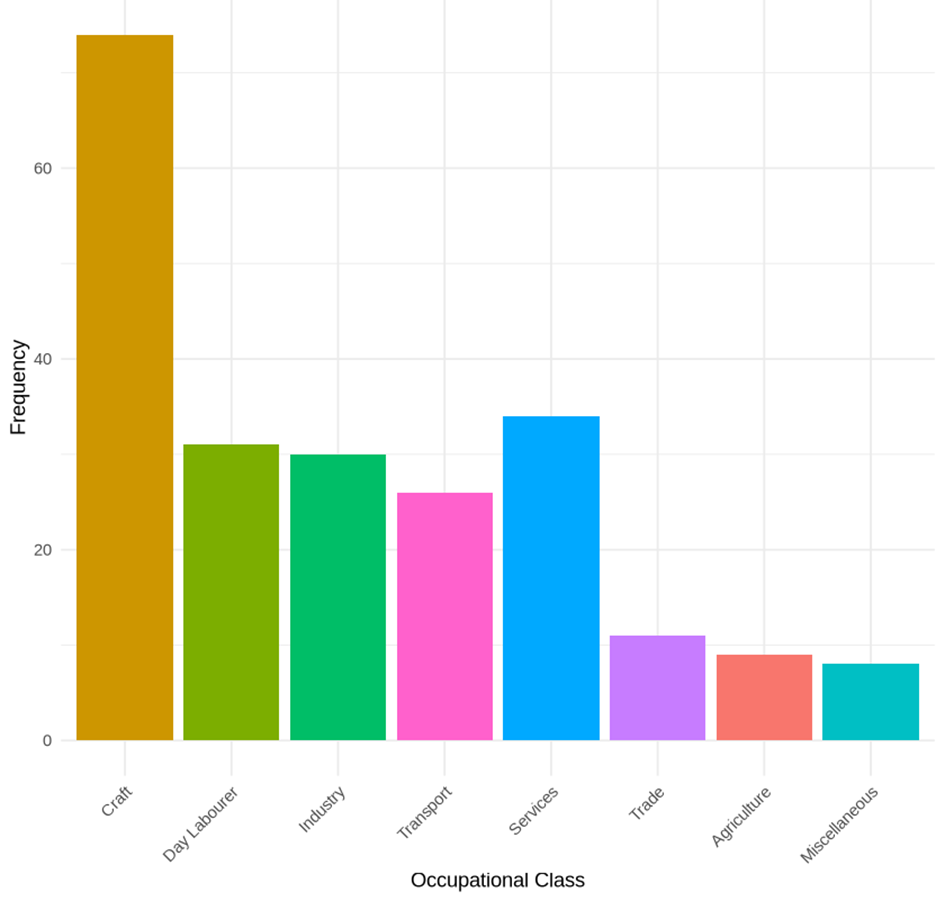


Fig. S5. Distribution of occupational classes.

The most named professions can be classified as craft with a wide range of craft occupations, with the most common of mason (14 individuals), locksmith (12), carpenter (9), blacksmith (6), and joiner (5), as well as brewer (3) and butcher (3). While for most individuals no specified information was given (39), for some of the individuals their grade was also stated, which related to a person’s experience and training in that craft. Among those with specified grades, master (11) was the most common designation, followed by helper (8) and apprentice (5). The second most frequently found occupation category was services. This term was more in the sense of public service and service occupations, and also included occupations that may fall more into the industrial sector, such as lithographic printers (4) and typesetters (1). Furthermore, three postmen and two each of accountants, bookkeepers, manservants, and messengers could be found here. Other occupations summarized here included street sweepers, barbers, caretakers, forest wardens, municipal field workers, and lecturers in mechanical engineering. A similar number of individuals were employed in occupations that can be categorized as industry or transportation. The transportation class most often includes pointsman (7), as well as relief signalman (4) or rolling stock technician (5); other occupations classified in this class include coachman, engine driver, railway head guard or stable hand for horse-drawn trams. The term industry was used to describe occupations that were clearly related to industrial production. These were mostly boilermakers (6 individuals), mechanics (4 individuals) or machine operators (3 individuals), otherwise factory worker, fitter, varnisher, but also weaver, stoker, operator, rubber worker or construction engineer are also classified here. Many occupations that are also practiced in industry can be found in both the day laborers and crafts classes. The second most frequently mentioned occupation was day laborer, while the third most frequently mentioned occupation was classified in the industry category. The term industry was used to describe occupations that were clearly related to industrial production. These were mostly boilermakers (6 individuals), mechanics (4) or machine operators (3), but otherwise factory workers, fitters, varnishers, weavers, stokers, operators, rubber workers, and construction engineers were also categorized here. However, it should be kept in mind that the other classes also included occupations that were carried out in industrial contexts. Few individuals can be assigned to the agriculture category alone. Although several individuals listed agriculture as an occupation, only few of them listed it as their only source of income and are summarized in this class. However, this category includes besides small farmers also three gardener helpers. The trade category includes several shop owners, as well as innkeepers and grocers, while the miscellaneous section includes for instance two men of private means, as well as house owner, Staff sergeant and Tripe-dresser.

The information about the workplace, which could be found in 48 individuals, provided more insight: Military service (9 individuals) and an association with the Railway System (38 individuals) where especially often named. While the individuals belonging to these classes were often classified in the transport class (such as pointsman or relief signalman), the classification into occupational classes was carried out without taking these details into account. Thus, also individuals with occupations from the craft sector (especially locksmiths), day laborers or industry can also be found showing an association with the Railway System. When the workplace is narrowed down even further, the Central Rail Maintenance Depot, the Krauss Locomotive Works or the Rail Maintenance Depot are named.

In 1828, Munich City Council established five classes of burials for adults (I–V) and three classes of burials for children (I–III), ranked from highest (Class I) to lowest (Class V). The highest burial class observed was Class III (n = 15), and only two individuals received a pauper’s burial, indicating publicly funded interment for impoverished individuals. Most individuals over 12 years were buried in Class V (n = 86) or Class IV (n = 66).


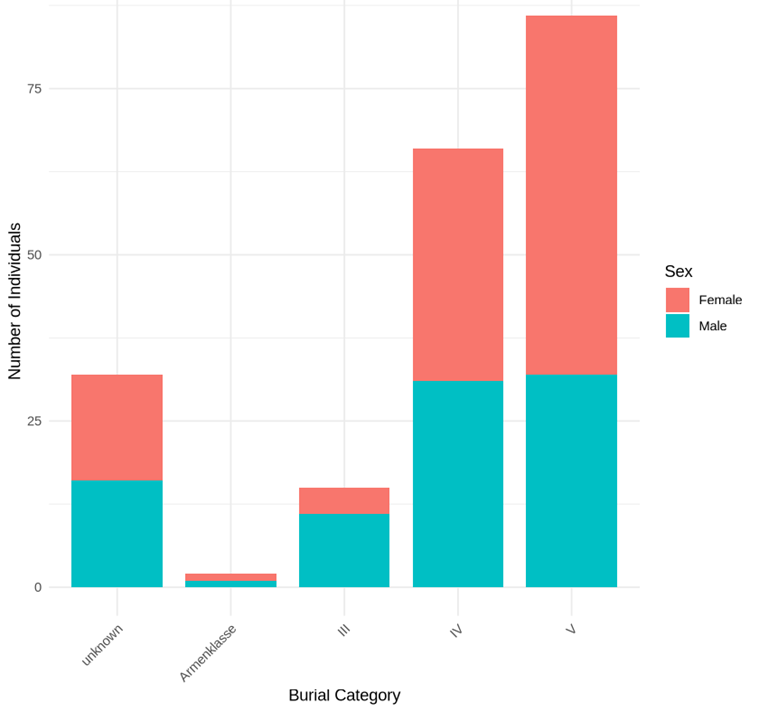


Fig. S6. Number of individuals per adult burial category by sex (Armenklasse = Pauper’s burial).

***Cause and circumstances of death – more details***

Causes of death were broadly categorized following the International Classification of Diseases, 11th Revision (ICD-11) (WHO 2019). Because 19th-century medical terminology differs substantially from modern clinical standards, causes of death were classified only at a broad level using overarching ICD-11 categories. This allowed grouping of causes into meaningful classes (e.g., infectious diseases, cardiovascular diseases, external causes) without overinterpreting ambiguous historical diagnoses.

Fig. S7 shows, that “certain infectious or parasitic diseases” is the by far the most common category, followed by “disease of the respiratory system”, “diseases of the circulatory system” and “neoplasms”.


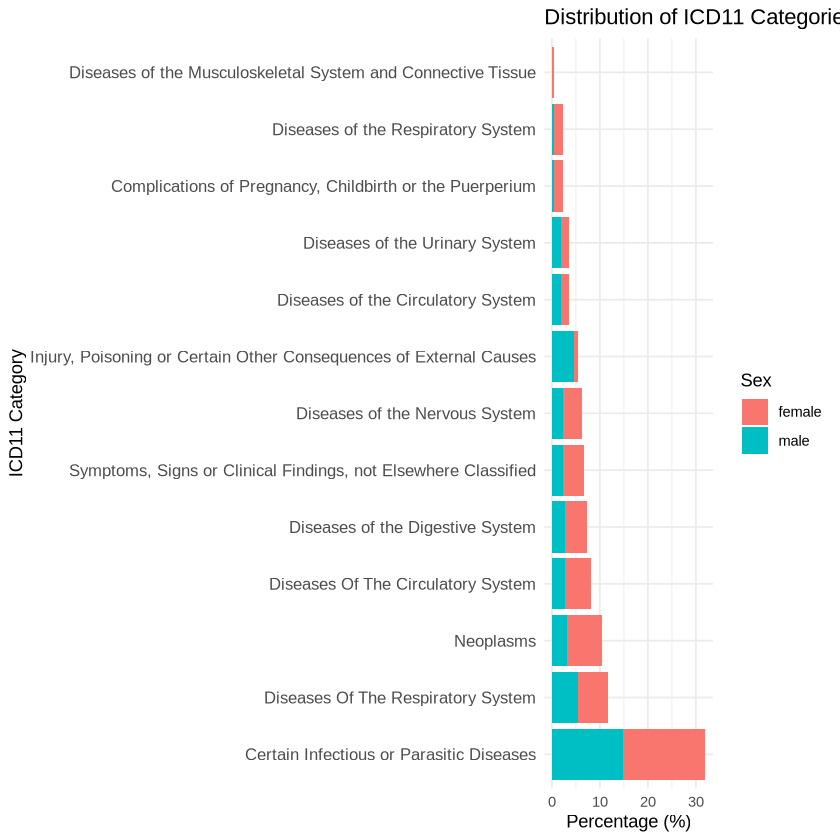


Fig. S7. Frequency of ICD-11 disease category.

**V The Skeletal remains**

***Extraskeletal elements***

Fig. S8–S12 show examples of mineralized extraskeletal elements.


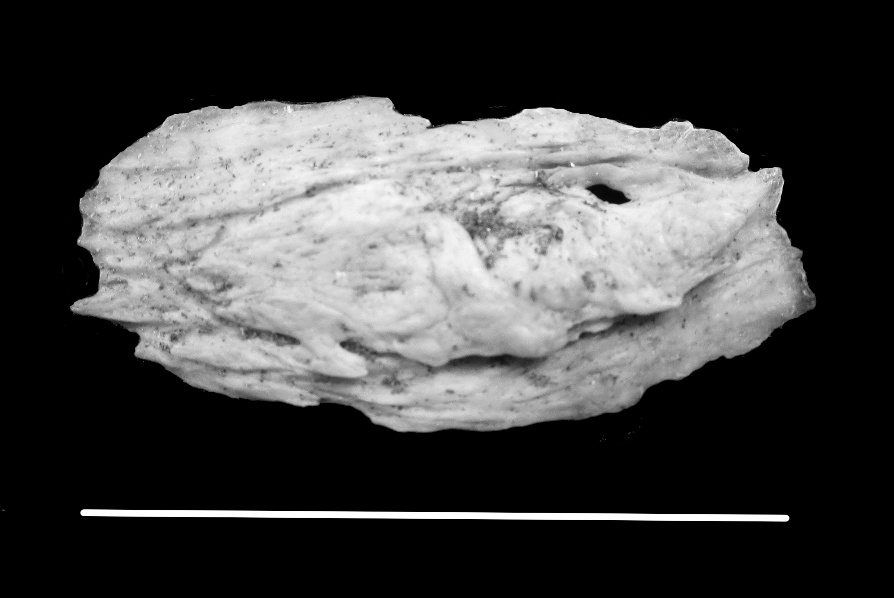


Fig. S8. Undefined, mineralized tissue with unknown in situ position from individual 82 (female; 65 years). Scale: 2 cm.


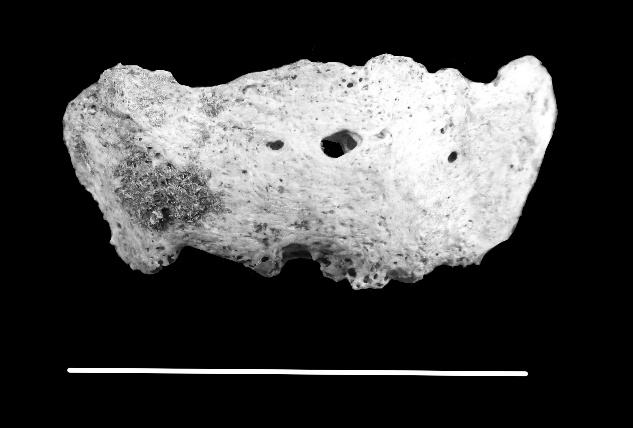

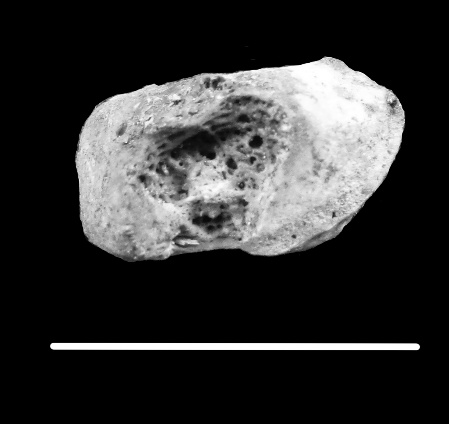


Fig. S9. Undefined, mineralized tissue with unknown in situ position from individual 89 (female; 51 years). Scale: 2 cm (left) and 3 cm (right).


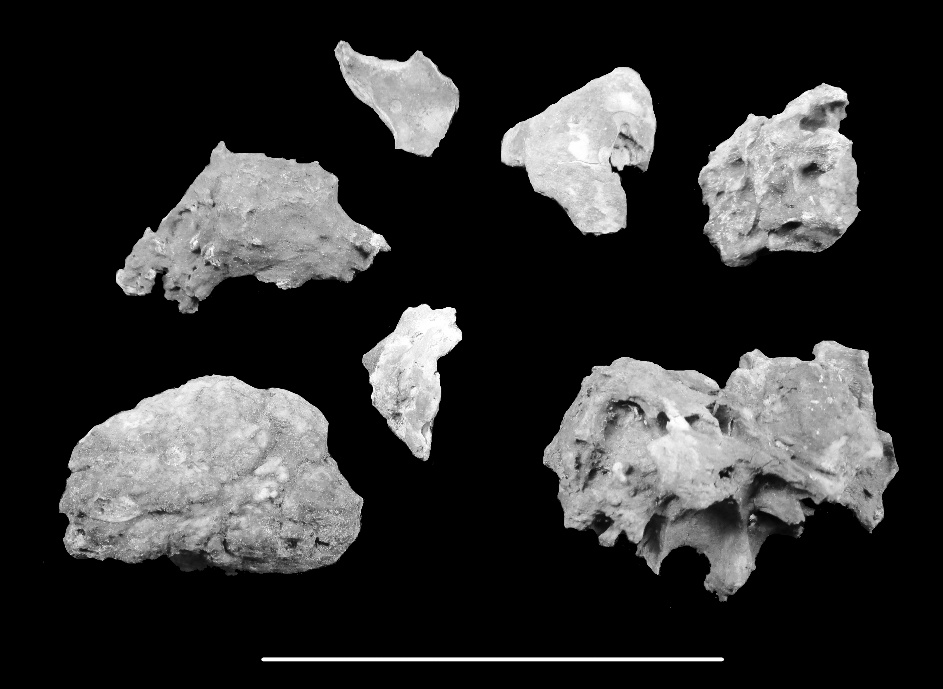


Fig. S10. Undefined, mineralized tissue found at the chest and throat region of individual 98 (female; 70 years). Scale: 1 cm.


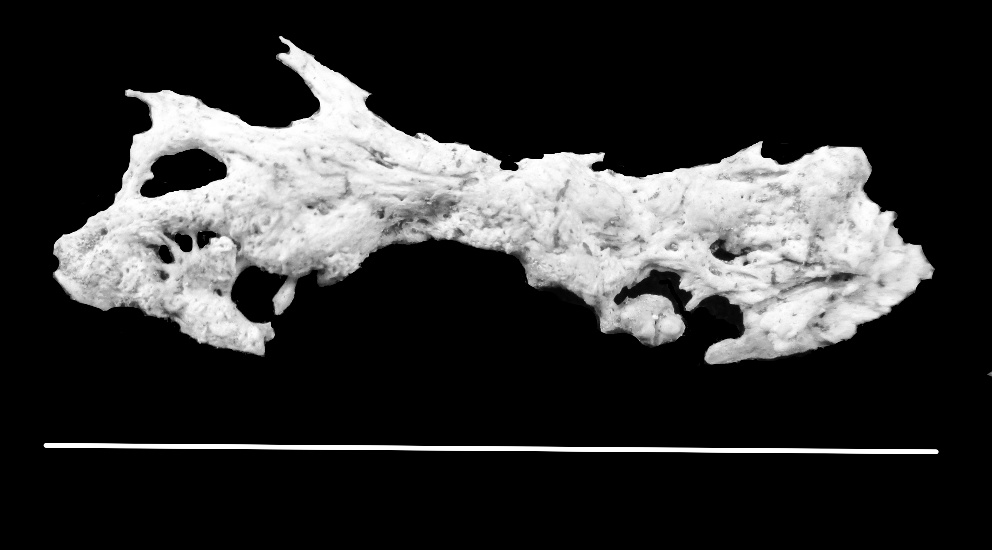


Fig. S11. Mineralized tissue with unknown in situ position of individual 121 (male; 52 years). Scale: 4 cm.


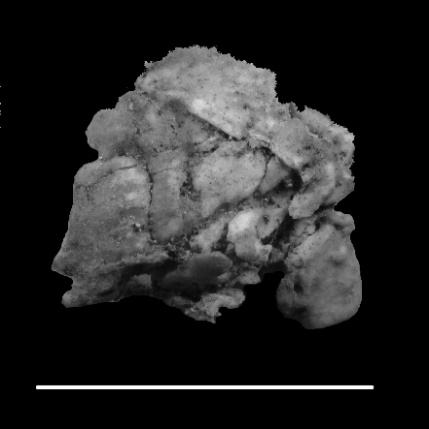

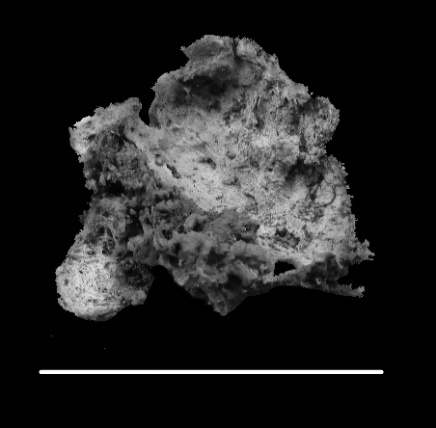


Fig. S12. Mineralized tissue found in the pelvis region of individual 150 (female; 46 years). Scale: 2 cm.

***Craniotomy***

Fig. S13 illustrates clear signs of craniotomy associated with a post-mortem examination.


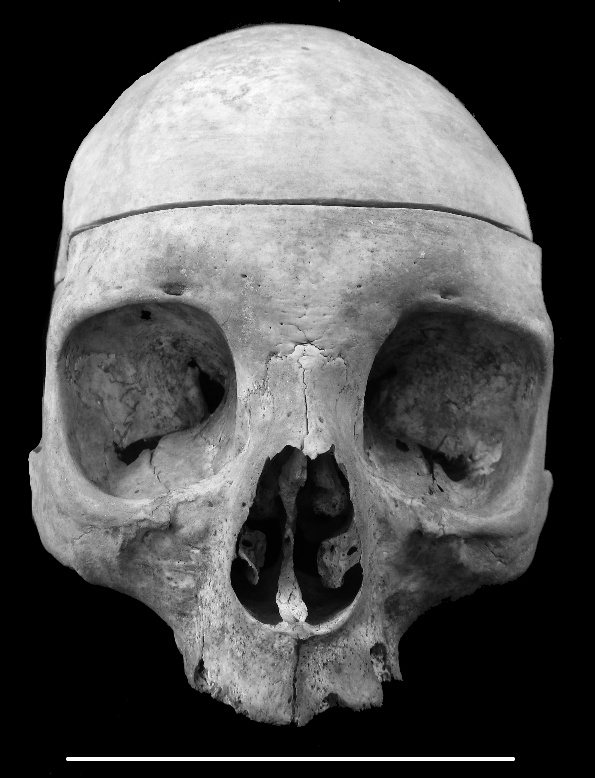

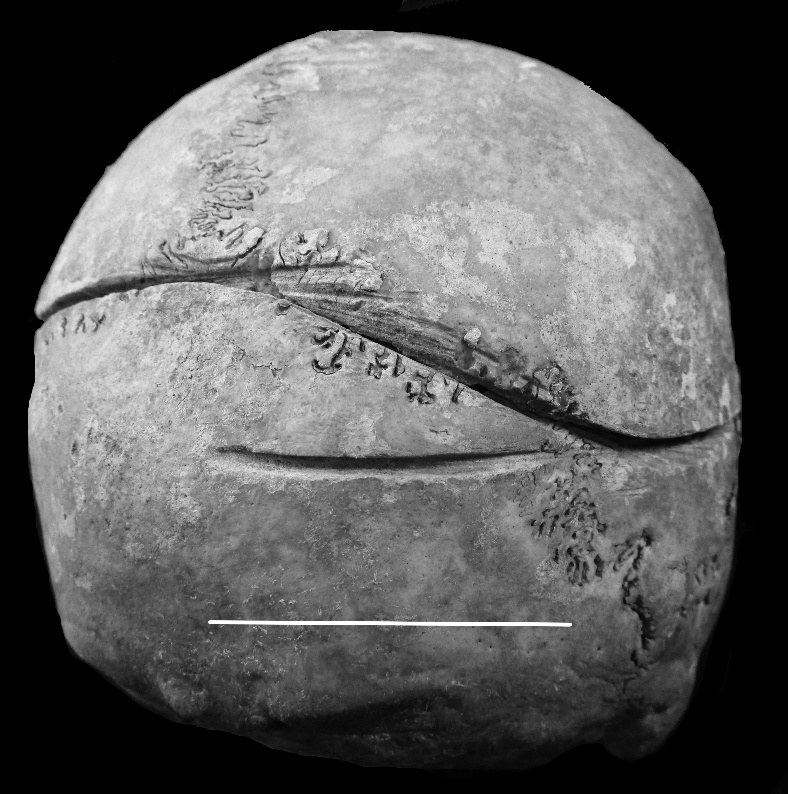

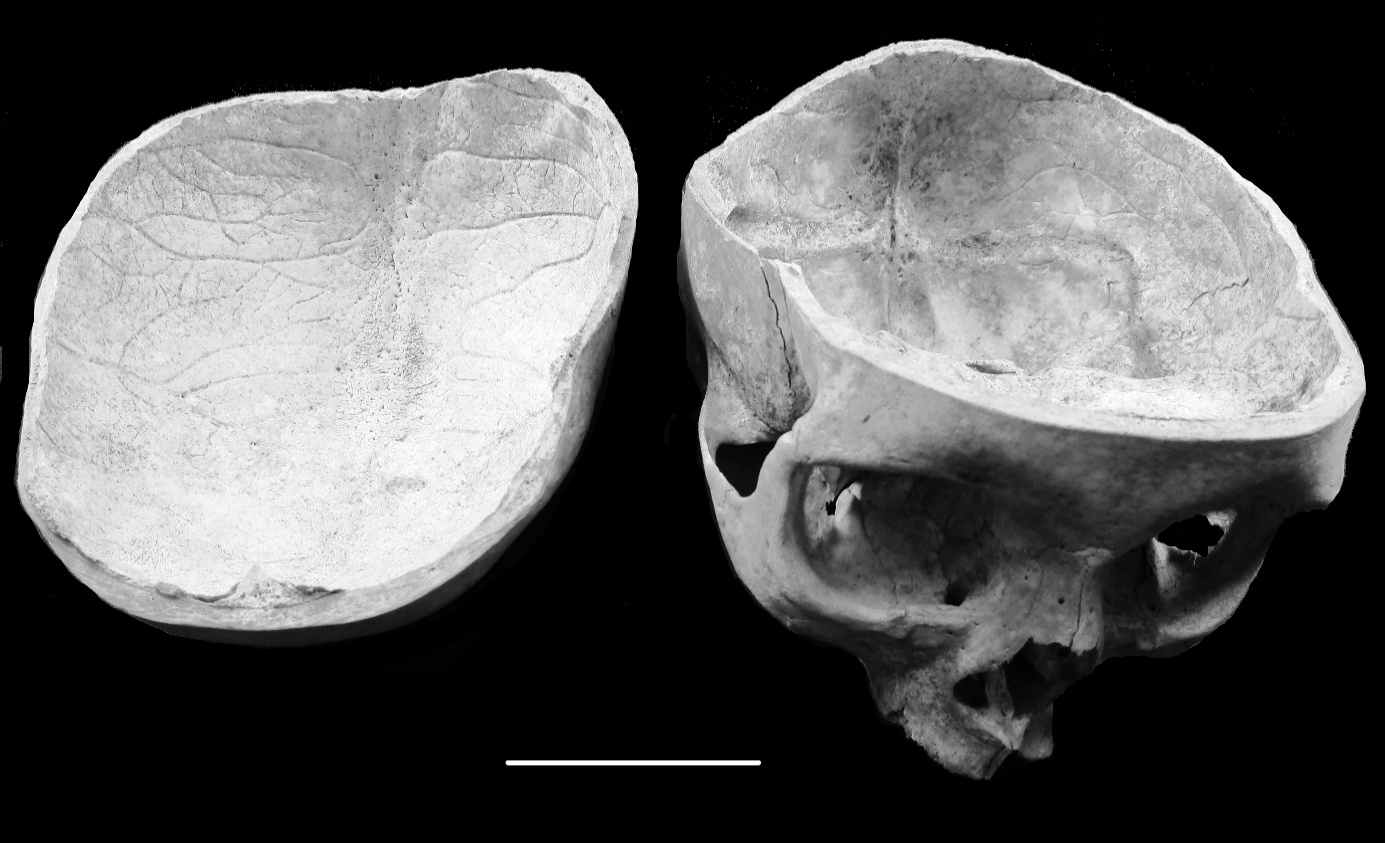


Fig. S13. Skull of individual 114 (female; 44 years) with signs of a postmortem autopsy. Scale: 5 cm.

**Literature**

Abegg, C., Hoxha, F., Campana, L., Ekizoglu, O., Schranz, S., Egger, C., Grabherr, S., Besse, M., & Moghaddam, N. 2023. Measuring pelvises in 3D surface scans and in MDCT generated virtual environment: Considerations for applications in the forensic context. *Forensic Science International*, 352, 111813. https://doi.org/10.1016/j.forsciint.2023.111813.

Aebi, T., d’Aujourd’hui, R., & Etter, H. F. 1989. Ausgrabungen in der Alten Stadtgärtnerei, Elsässerstrasse 2a (St. Johann Park). In *Jahresbericht der Archäologischen Bodenforschung des Kantons Basel-Stadt,* 1989: 206–245. Werner Druck. https://doi.org/10.12685/jbab.1989.206-249.

Allemann, M., Bernasconi, M., Spichtig, N., & Steiner, S. 2016. Ausgrabung und Funde im Jahre 2015. In *Jahresbericht der Archäologischen Bodenforschung Basel-Stadt,* 2015: 39–79. Stadt Basel. https://doi.org/10.12685/JBAB.2015.39-79.

AlQahtani, S. J., Hector, M. P., & Liversidge, H. M. 2010. Brief communication: The London atlas of human tooth development and eruption. *American Journal of Physical Anthropology*, 142: 481–490. https://doi.org/10.1002/ajpa.21258.

Baker, B. J., Dupras, T. L., & Tocheri, M. W. 2005. *The Osteology of Infants and Children*. Texas A&M University Press.

Buckberry, J. L., & Chamberlain, A. T. 2002. Age estimation from the auricular surface of the ilium: a revised method. *American Journal of Physical Anthropology*, 119: 231–239. https://doi.org/10.1002/ajpa.10130.

Grupe, G., Harbeck, M., & McGlynn, G. 2015. *Prähistorische Anthropologie*. Springer-Verlag Berlin, Heidelberg.

Harbeck, M., & von Heyking, K. 2023. Standardisierte Skelettdokumentation: Vorgabe für Körpergräber. SNSB – State Collection for Anthropology Munich. https://sam.snsb.de/wp-content/uploads/sites/9/2023/09/27456adf.pdf.

Harbeck, M., & Paulus, C. (eds.). In press. *The Winthir Collection: A documented osteological collection from Central Europe*. Sidestone Press, Leiden. https://doi.org/10.59641/g7j3d4e5f6.

Heeb, B., & Teßmann, B. 2021. Zur Geschichte der anthropologischen Rudolf-Virchow-Sammlung (RV-Sammlung). In *Annals of the History and Philosophy of Biology,* 24 (2019), Deutsche Gesellschaft für Geschichte und Theorie der Biologie (ed.): 75–90. Göttingen University Press. https://doi.org/10.17875/gup2021-1583.

Herrmann, B., Grupe, G., Hummel, S., Piepenbrink, H., & Schutkowski, H. (eds.). 1990. *Prähistorische Anthropologie. Leitfaden der Feld- und Labormethoden*. Springer-Verlag, Berlin, Heidelberg, New York.

Hotz, G., Zulauf-Semmler, M., & Fiebig-Ebneter, V. 2016. Der Spitalfriedhof und das Bürgerspital zu Basel: Anthropologie und Geschichtswissenschaften am Beispiel des Spitalfriedhofs. In *Jahresbericht der Archäologischen Bodenforschung Basel-Stadt*, 2015: 122–131. https://doi.org/10.12685/jbab.2015.122-131.

Indra, L., Vach, W., Desideri, J., Besse, M., & Pichler, S. L. 2021. Testing the validity of population-specific sex estimation equations: An evaluation based on talus and patella measurements. *Science & Justice*, 61(5): 555–563. https://doi.org/10.1016/j.scijus.2021.06.011.

Nemeskéri, J., Harsányi, L., & Acsádi, G. 1960. Methoden zur Diagnose des Lebensalters von Skelettfunden. *Anthropologischer Anzeiger*, 24: 70–95.

Petaros, A., Caplova, Z., Verna, E., Adalian, P., Baccino, E., de Boer, H. H., Cunha, E., Ekizoglu, O., Ferreira, M. T., Fracasso, T., Kranioti, E. F., Lefevre, P., Lynnerup, N., Ross, A., Steyn, M., Obertova, Z., & Cattaneo, C. 2021. Technical Note: The Forensic Anthropology Society of Europe (FASE) Map of Identified Osteological Collections. *Forensic Science International*, 328, 110995. https://doi.org/10.1016/j.forsciint.2021.110995.

Rühli, F. J., Hotz, G., & Böni, T. 2003. Brief communication: The Galler Collection: A little-known historic Swiss bone pathology reference series. *American Journal of Physical Anthropology*, 121(1): 15–18. https://doi.org/10.1002/ajpa.10219.

Salega, S., & Grosskopf, B. 2022. Evaluation of entheseal changes in a modern identified skeletal collection from Inden (Germany). *International Journal of Osteoarchaeology*, 32(1): 86–99. https://doi.org/10.1002/oa.3046.

Schaefer, M., Black, S., & Scheuer, L. 2009. *Juvenile Osteology: A Laboratory and Field Manual*. Academic Press, Burlington, San Diego, London.

Scheuer, L., & Black, S. 2000. *Developmental Juvenile Osteology*. Academic Press, San Diego, London.

Stoecker, H., Schnalke, T., & Winkelmann, A. (eds.). 2013. *Sammeln, Erforschen, Zurückgeben?: Menschliche Gebeine aus der Kolonialzeit in akademischen und musealen Sammlungen (1st edition)*. Ch. Links Verlag, Berlin.

Stremke, F., Schreil, F., & Harbeck, M. In press. Excavation and structure of the archaeological site. In *The Winthir Collection: A documented osteological collection from Central Europe*, Harbeck, M. & Paulus, C. (eds.). Sidestone Press, Leiden. https://doi.org/10.59641/g7j3d4e5f6.

Szikossy, I., Bernert, Zs., & Pap, I. 1997. Mummies from the 18th–19th century Dominican church of Vác, Hungary. *Acta Biologica Szeged*, 42: 145–150.

Ubelaker, D. H. 1978. Human Skeletal Remains: Excavation, Analysis, Interpretation. *American Anthropologist*, 81(2): 446–448.
